# Cytotoxicity of silver and copper nanoparticles on rainbow trout (*Oncorhynchus mykiss*) hepatocytes

**DOI:** 10.1007/s11356-017-0494-0

**Published:** 2017-10-25

**Authors:** Teresa Ostaszewska, Jerzy Śliwiński, Maciej Kamaszewski, Paweł Sysa, Maciej Chojnacki

**Affiliations:** 1 Department of Ichthyobiology, Fisheries and Aquaculture Biotechnology, Faculty of Animal Science, Warsaw University of Life Sciences, Ciszewskiego 8, 02-786 Warsaw, Poland; 2 Center of Veterinary Medicine, Nicolaus Copernicus University in Toruń, Gagarina 11, 87-100 Toruń, Poland

**Keywords:** Copper nanoparticles, Hepatocytes, Histopathology, Rainbow trout, Silver nanoparticles, Ultrastructure

## Abstract

Nanoparticles are commonly used in the industry and are present in consumer goods; therefore, evaluation of their potential toxicity is necessary. The aim of the present study was to assess the cytotoxic effects of the nanoparticles of silver (AgNPs) at the concentration of 1.5 mg L^−1^ and copper (CuNPs) at 0.15 mg L^−1^ on rainbow trout (*Oncorhynchus mykiss*) hepatocytes after 28 days of exposure. Histological analysis revealed dilated sinusoids, shrunken hepatocytes, nuclear necrosis, and increased number of Kupffer cells in the liver of fish exposed to nanoparticles. The lowest hepatocyte proliferation index was observed in fish treated with AgNPs. Ultrastructural studies revealed mitochondrial edema and cristolysis, dilated and loosened endoplasmic reticulum, cytoplasm vacuolation, accumulation of lipid droplets, glycogen depletion, and formation of myelin-like bodies. The results also revealed that the liver of fish exposed to copper nanoparticles showed higher regenerative potential indicated by higher proliferation index, more abundant glycogen, and more numerous Kupffer cells compared to the fish treated with silver nanoparticles.

## Introduction

The use of nanoparticles in various industries is increasing rapidly due to their unique and beneficial physicochemical properties attributed to their small size, chemical composition, surface structure, solubility, shape, and aggregation (Nel et al. 2006). From an ecotoxycological point of view, nanoparticles in the aqueous suspensions represent as a solid phase similar to that of poorly soluble compounds, and a wide range of processes like dissolution, agglomeration/aggregation, sedimentation, surface transformation, and some chemical reactions can result in their dynamic exposure concentration (Skjolding et al. 2016).

Nanoparticles may considerably affect ecosystems and thus the health of animals and humans (Handy et al. 2008). The increasing use of nanoparticles in the industry and consumer goods brings about the need for risk assessment and evaluation of the effects of nanoparticles on human health and natural environment (Schrand et al. 2010). The production and use of nanoparticles are not yet regulated by international safety regulations (EC 2008) despite many proposals.

In aquatic environment, nanoparticles accumulate, aggregate, and then precipitate to bottom sediments (Farré et al. 2009). In some rivers, the concentration of nanocopper and nanosilver reached 0.06 and 0.04 mg L^−1^, respectively (Chio et al. 2012).

The toxicity of nanoparticles depends on their type, shape, size, surface area and time of their retention in the organism, metabolism, and biodistribution. Xue et al. (2012) intravenously injected mice with Ag nanoparticle (size, 21.8 nm) saline suspension and observed its migration to various tissues, mainly to the spleen and liver. It is suggested that the liver, being a key detoxication organ, is involved in nanoparticle elimination and may transitorily accumulate higher concentrations of these particles, as it was shown for Nile tilapia diet treated with TiO_2_ nanoparticles (size, 24.1 nm) (Ramsden et al. 2009). Farkas et al. (2010) revealed that Ag nanoparticles (size, 1–10 nm) were highly cytotoxic to rainbow trout hepatocytes which was manifested by the reduction of metabolic activity and membrane integrity, while Au nanoparticles (size, 5–10 nm) caused a threefold elevation of ROS levels. Hepatocytes of the juvenile carp exposed to TiO_2_ nanoparticles (size, 50 nm) showed cytoplasm vacuolation and apoptosome, including necrotic cell bodies and apoptotic-like bodies, and a few foci of lipidosis with fatty change (Hao et al. 2009). Similarly, a wide range of histopathological anomalies was observed in the hepatocytes of Siberian sturgeon larvae treated with Ag nanoparticles (size, 8.02 nm) and Cu nanoparticles (size, 10.24 nm) including hepatocyte shrinkage, vacuolization, and pycnotic nuclei (Ostarszewska et al. 2016).

However, little data is available in the literature on the effects of nanoparticles on hepatocyte ultrastructure in fish; this issue was studied only in common carp (*Cyprinus carpio*) and Nile tilapia (*Oreochromis niloticus*) subjected to nanoparticles (Lee et al. 2012; Alkaladi et al. 2014). No studies of the toxic impact of Ag or Cu nanoparticles on the ultrastructure of salmonid hepatocytes have been performed. The aim of the present study was to evaluate cytotoxic alterations induced by copper and silver nanoparticles in the hepatocytes of rainbow trout (*Oncorhynchus mykiss*).

## Materials and methods

### Nanoparticles (AgNPs and CuNPs) used in the experiment

Nanosilver (size < 100 nm, surface area 5.0 m^2^ g^−1^, density 10.49 g cm^−1^, and purity of 99.5%) and nanocopper (size < 50 nm, density 8.94 g cm^−1^, and purity of 99.5%) powders were purchased from Sigma Aldrich, UK (cat. no 684007 and 684007, respectively). Both nanoparticle stocks (50 mg L^−1^) were suspended in Milli-Q water and sonicated for 30 min (sonicator, 250 W, 40 kHz, 25 °C; Ultron U-507 Ultron, Poland). After sonication, the suspensions were filtered through the 200-nm nylon membrane (Whatman®, UK). The average size of Ag and Cu nanoparticles in stock suspension, measured by transmission electron microscopy (TEM) (JEOL JEM-1220 TE microscope, JEOL Ltd., Japan), was 8.02 ± 2.49 and 10.24 ± 1.99 nm, for Ag and Cu nanoparticles, respectively, and the aggregates of average size 235.5 ± 25.1 nm for Ag nanoparticles and 338.0 ± 55.8 nm for Cu nanoparticles were observed. The zeta potential, evaluated by dynamic light-scattering (DLS) method on Zetasizer Nano-ZS90 (Malvern, Worcestershire, UK), for Ag nanoparticles was 53.6 ± 5.0 mV and for Cu nanoparticles was 29.5 ± 0.7 mV. Each sample was measured after 120 s of stabilization at 25 °C, pH 8.6, in 20 replicates (Ostaszewska et al. 2016).

### Experimental design

Rainbow trout of 23.57 ± 1.15-mm body length and 0.12 ± 0.02-g body mass used in the experiment were obtained from the fish farm in Boży Dar, Poland, and transported to the Department of Ichthyobiology, Fisheries and Aquaculture Biotechnology of Warsaw University of Life Sciences. Based on the results of the acute toxicity test (96 h) (LC50, 17.5 and 2.00 mg L^−1^ for Ag and Cu nanoparticles, respectively), concentrations of 1.5 mg L^−1^ (Ag) and 0.15 mg L^−1^ (Cu) nanoparticles were used. The experiment was carried out for 28 days in 9 tanks of 20-L volume, with fish density of 2.5 ind. L^−1^ for Ag and Cu nanoparticles and the control group in triplicates. The mean water temperature in experimental tanks was 18.25 ± 0.77 °C, pH 8.4 ± 0.18, and the concentration of dissolved oxygen was 8.70 ± 0.25 mg L^−1^. Daily, 80% of tank water was changed, with nanoparticle concentration maintenance on the same level. Photoperiod was set at 12-h light/12-h dark. For the first 6 days of the experiment, the trout were fed natural food—*Artemia* sp. *nauplii* (IchthyoTrophic, Poland)—ad libitum and then from the 7th day commercial diet Larva Proactive (Skretting, Norway). During the experiment, dead fish were removed and counted. Survival was calculated based on cumulative mortality and was shown in percent. On the last day of the experiment, the body length and mass of the fish (15 from each group) were measured, with accuracy 0.01 mm (length) and 0.01 g (mass). Before measurements, fish were anesthetized with buffered MS-222 (ethyl 3-aminobenzoate methanesulfonic acid), solution 1:5000, of pH 7.5. Tissues were collected from 15 individuals from each experimental and control group.

For light microscope analysis, the tissues were preserved in Bouin’s solution or 4% paraformaldehyde, rinsed after 24 h, and dehydrated in ethanol solutions of increasing concentration. Then, the preparations were cleared with xylene and embedded in paraffin (Leica Microsystems, Germany). The paraffin blocks were cut into 5-μm-thick sections using Leica RM 2265 rotary microtome (Leica Microsystems, Germany) and stained with hematoxylin-eosin (H-E), alcian blue, and Schiff’s reagent (alcian blue 8GX pH 1.0, pH 2.5, Schiff’s reagent—AB-PAS) (Pearse 1985).

Immunohistochemical analysis was performed using hepatic tissue sections preserved with Bouin’s solution in order to evaluate cell proliferation (staining for proliferating cell nuclear antigen—PCNA), according to the method described by Ostaszewska et al. (2008).

Proliferation index (percentage of PCNA-positive nuclei in relation to all hepatocyte cell nuclei) was calculated in 20 fields of 35,000 μm^2^ for 15 fish from each experimental group. Morphometric analysis was done for 20 randomly selected sections of 15 fish from each experimental group.

Material for ultrastructural analysis was preserved in 2.5% glutaraldehyde solution. Then, the samples were treated with 1% osmium tetroxide and embedded in Epon 812 (Electron Microscopy Sciences, USA). Ultrathin sections were contrasted with uranyl acetate and lead citrate. Observations and electron micrographs were done using electron transmission microscope Joel JEM 100 C (JEOL Ltd., Japan). Measurements of cellular structures were made in the micrographs: hepatocyte surface area, diameter of hepatocyte nuclei, number and area of mitochondria and peroxisomes, and area occupied by lipids and glycogen (measurements were done in 20 fields for 15 fish from each experimental group).

### Statistical analysis

Fish survival, body mass and length, and cellular structures were analyzed with one-way ANOVA followed by Tukey’s post hoc test (*p* ≤ 0.05) (Statistica 12.0, StatSoft Inc., OK, USA). The results of all measurements were presented as mean ± standard deviation (SD).

## Results

### Survival and growth rate of rainbow trout

Survival of fish from the control group was higher compared to the fish intoxicated with AgNP and CuNP solutions (Table 1). The lowest survival was observed in the CuNP group (Table 1). The fish from this group showed also the lowest body mass and length.

**Table 1:** Survival and growth of rainbow trout in the control and in the groups exposed for 28 days to silver (1.5 mg L^−1^) or copper (0.15 mg L^−1^) nanoparticles (*n* = 3) (mean ± SD)

Parameter	Control	AgNPs	CuNPs
Survival (%)	99.26 ± 2.02^a^	97.56 ± 0.33^a^	85.50 ± 1.33^b^
Body weight (g)	0.97 ± 0.09^a^	0.72 ± 0.11^ab^	0.64 ± 0.05^b^
Body length (mm)	39.06 ± 3.12	35.4 ± 3.20	34.23 ± 1.32

Different lowercase letters indicate statistical differences between groups (*p* ≤ 0.05)

### Histological analyses of rainbow trout hepatic tissue

**Fig. 1:**
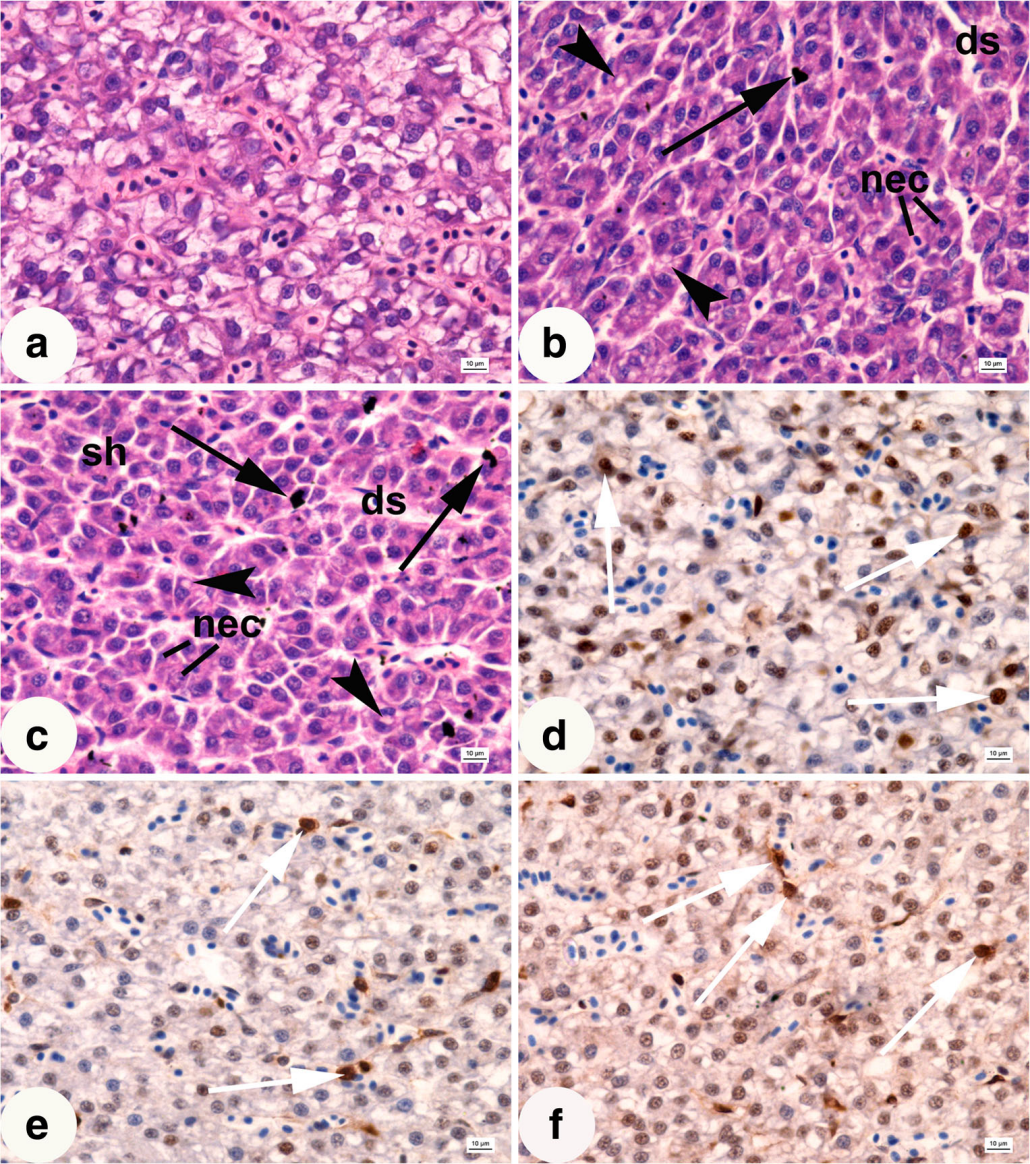
Sections of rainbow trout liver after 28 days of exposure. **a** Control. **b** Fish exposed to 1.5 mg L^−1^ of silver nanoparticles. **c** Fish exposed to 0.15 mg L^−1^ of copper nanoparticles. **a–c** H-E staining. **d** Control. **e** Fish exposed to 1.5 mg L^−1^ of silver nanoparticles. **f** Fish exposed to 0.15 mg L^−1^ of copper nanoparticles. **d–f** PCNA staining. nec, necrosis; ds, dilated sinusoids; arrowhead, hepatocyte vacuolation; black arrows, Kupffer cells; white arrows, PCNA-positive nuclei; scale bar = 10 μm

In the control group, light microscope analysis revealed large polygonal hepatocytes (Table 2) showing distinct oval cell nuclei with one or more nucleoli. Hepatocytes were situated around the bile canaliculi and along sinusoids (Fig. 1a and Table 2). They showed large distinctly stained glycogen areas separated from the perinuclear zone containing organelles and lipid droplets (Fig. 1a). The hepatic tissue showed also Kupffer cells. Hepatocytes of fish exposed to silver and copper nanoparticles presented smaller area and showed lower proliferation index compared to the control (Table 2). The lowest proliferation index was observed in the liver of fish exposed to silver nanoparticles (Fig. 1d–f and Table 2). Hepatocyte vacuolation occurred in fish exposed to silver and copper nanoparticles (Fig. 1b, c and Table 2). In the liver of fish exposed to nanoparticles, histopathological alterations were observed, dilated sinusoids, necrosis, vacuolation and shrinkage of hepatocytes, pyknosis of nuclei, and increase in Kupffer cell abundance, compared to the control (Fig. 1a–c). The highest density of Kupffer cells occurred in the liver of fish treated with copper nanoparticles (Table 2).

**Table 2:** The results of histomorphometric measurements of the hepatic tissue of rainbow trout from the control group and that exposed to silver (1.5 mg L^−1^) and copper (0.15 mg L^−1^) nanoparticles after 28 days of treatment (*n* = 100) (mean ± SD)

Parameter	Control	AgNPs	CuNPs
Area of hepatocytes (μm^2^)	113.46 ± 9^a^	85.22 ± 14^ab^	79.86 ± 11^b^
Diameter of hepatocytes nuclei (μm)	6.1 ± 0.67	5.03 ± 0.99	5.39 ± 0.68
Number of Kupffer cells (in 1000 μm^2^)	0.04 ± 0.03^a^	0.15 ± 0.07^ab^	0.26 ± 0.11^b^
Proliferative index (%)	60.91 ± 2.21^a^	30.37 ± 3.18^c^	52.22 ± 3.26^b^

Different lowercase letters indicate statistical differences between groups (*p* ≤ 0.05)

**Fig. 2:**
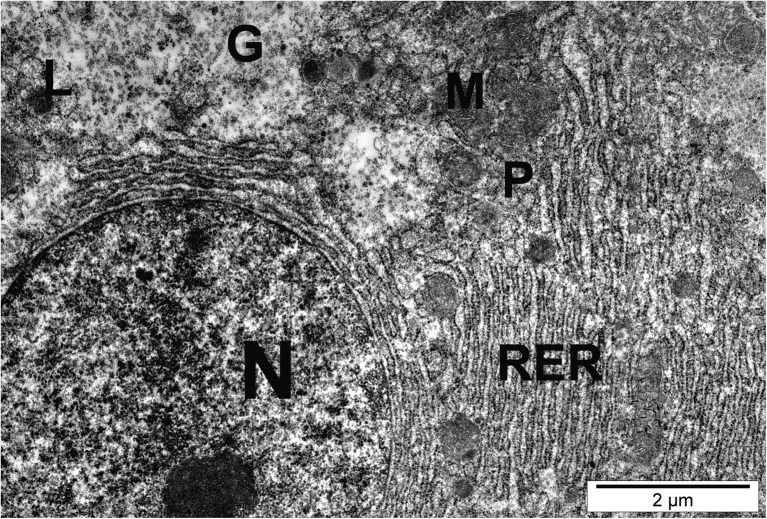
Control hepatocyte showing normal organelle ultrastructure with rounded nucleus (N), numerous mitochondria (M), rough endoplasmic reticulum (RER), peroxisomes (P), glycogen particles (G), and lipid droplets (L)

Electron microscope analysis revealed regular location of cell components in the control group (Fig. 2). Hepatocyte nuclei showed scarce chromatin scattered in the karyoplasm. The nucleoli were homogenous and showed high electronic density. Rough endoplasmic reticulum (RER) cisternae surrounded the centrally located nucleus. They showed no fragmentation and were organized in layers of 14–16. The RER envelope showed numerous mitochondria and peroxisomes. Mitochondria with numerous cristae were oval or elongated. Peroxisomes were located at the outer border of the RER envelope, usually near mitochondria. Lysosomes, the Golgi apparatus, and smooth endoplasmic reticulum (SER) were located between the nucleus and bile canaliculi. Lysosomes were scarcely distributed near the bile canaliculi. Golgi apparatuses consisted of three to five cisternae forming numerous vesicles containing low-electron-density lipoproteins. SER was visible as an irregular network of tubular profiles (Fig. 2).

**Fig. 3:**
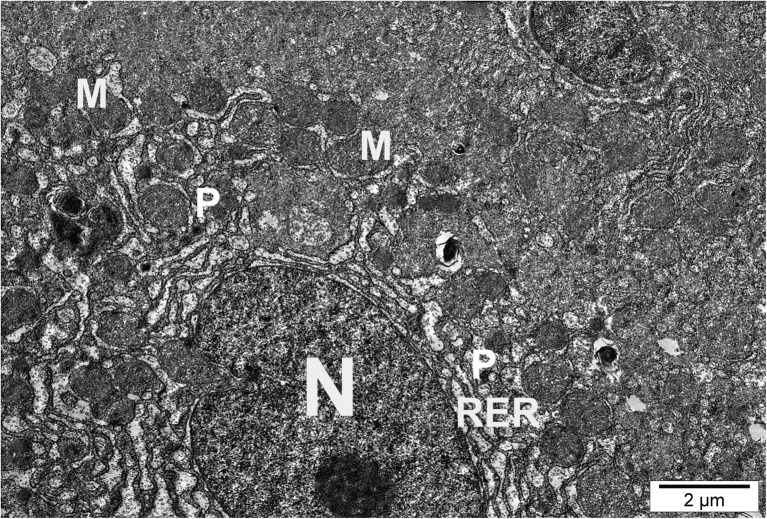
Hepatocyte of fish exposed to silver nanoparticles. Nuclear degradation (N), RER dilatation (RER), peroxisomes (P), and numerous mitochondria (M) were visible

Electron microscope analysis revealed considerable cytopathological alterations in hepatic tissue of fish exposed to silver and copper nanoparticles compared to the control group. Distinct disorganization of organelles was observed accompanied by glycogen depletion (Table 3). Organelles were irregularly distributed within the cytoplasm. In the RER, disruption and reduction of profiles and loss of stacks accompanied by shortening, dilatation, loosening, and degranulation of RER and ribosome loss were observed (Fig. 3).

**Table 3:** The results of histomorphometric measurements of cell organelles in the hepatocytes of rainbow trout from the control group and those exposed to silver (1.5 mg L^−1^) and copper (0.15 mg L^−1^) nanoparticles after 28 days of treatment (*n* = 100) (mean ± SD)

Parameter	Control	AgNPs	CuNPs
Area occupied by mitochondria per 1000 μm^2^	3.95 ± 0.45^a^	4.64 ± 0.74^a^	4.51 ± 1.25^a^
Number of mitochondria per 1000 μm^2^	168 ± 17.1^a^	212.7 ± 11.2^ab^	252.8 ± 34.6^b^
Area occupied by peroxisomes per 1000 μm^2^	0.74 ± 0.04^a^	0.84 ± 0.13^a^	0.81 ± 0.02^a^
Area occupied by lipid droplets per 1000 μm^2^	3.54 ± 0.31^a^	5.23 ± 1.36^a^	4.77 ± 0.80^a^
Area occupied by glycogen per 1000 μm^2^	12.70 ± 2.33^c^	1.20 ± 0.18^a^	5.95 ± 0.76^b^

Different lowercase letters indicate statistical differences between (*p* ≤ 0.05)

**Fig. 4:**
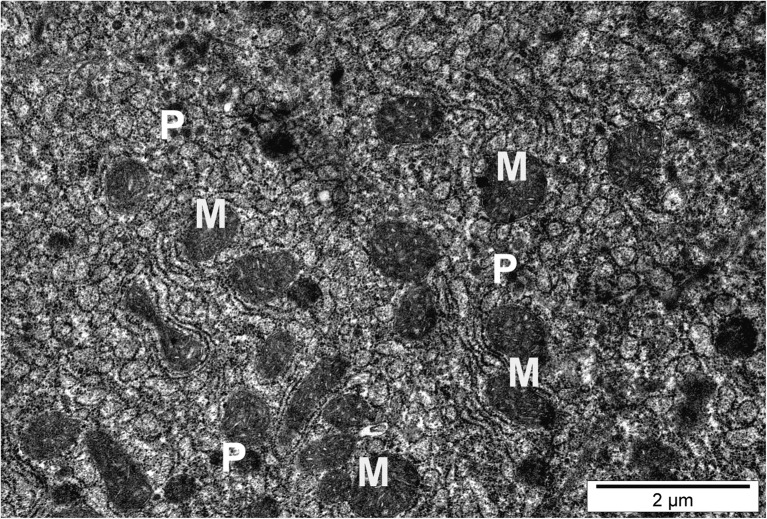
Hepatocyte of fish exposed to silver nanoparticles. Numerous mitochondria (M) of different shapes and sizes. P, peroxisomes

Mitochondria showed in homogenous structure in size and morphology. Pleomorphic mitochondria showed edema, cristolysis, curvatures, and elongation. Mitochondria were in close connection with peroxisomes. In hepatocytes of fish intoxicated with copper and silver nanoparticles, the area occupied by peroxisomes and mitochondria slightly increased (Fig. 4 and Table 3).

**Fig. 5:**
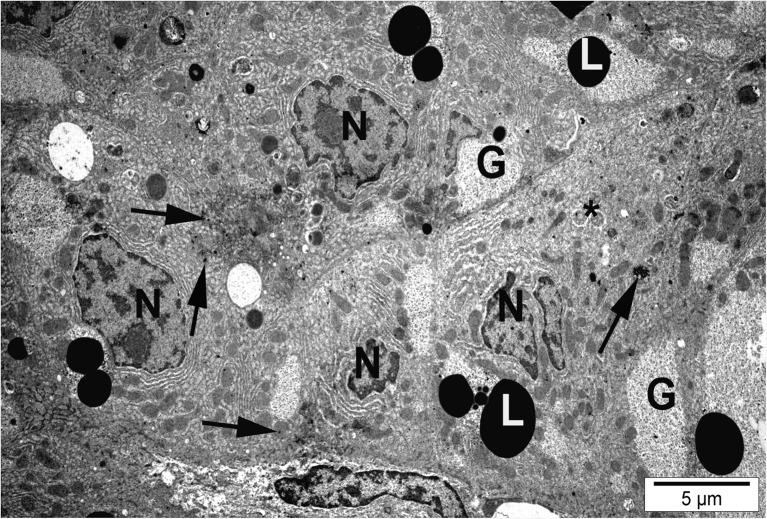
Hepatocyte of fish exposed to copper nanoparticles. N, nuclear degradation; L, numerous lipid droplets; G, glycogen; *, autophagosomes; black arrows, particles of nanocopper

Cytoplasm contained more abundant lipid droplets of various sizes compared to the control (Fig. 5 and Table 3).

**Fig. 6:**
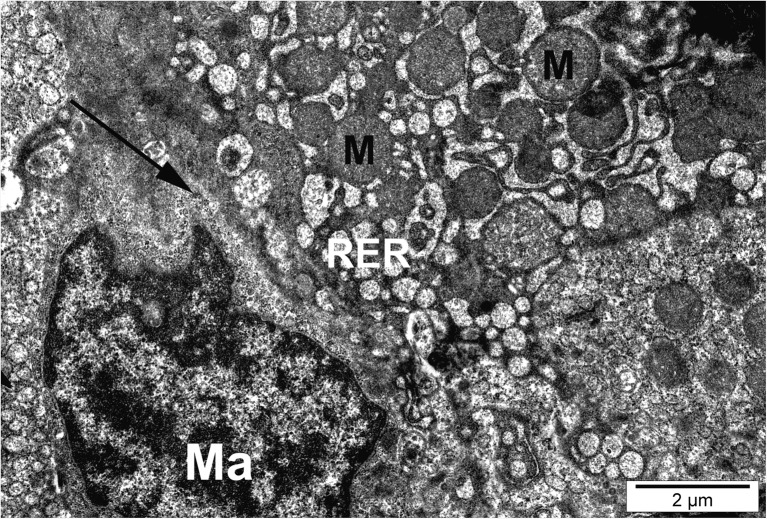
Hepatocyte of fish exposed to silver nanoparticles. Necrotic cell with dilated endoplasmic reticulum (RER), swollen mitochondria (M), and macrophage (Ma) with particles of nanosilver (black arrow)

**Fig. 7:**
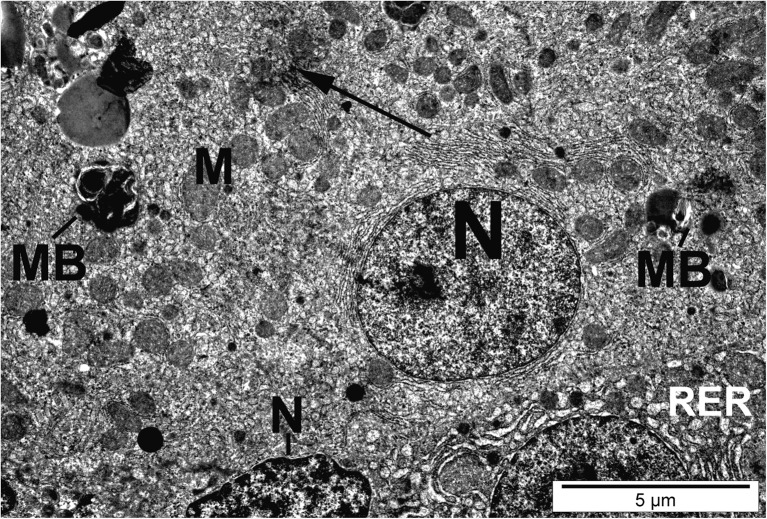
Hepatocytes of fish exposed to silver nanoparticles. Normal and necrotic cells with dilated endoplasmic reticulum (RER), myelin-like bodies (MB), and particles of nanosilver (black arrow). M, mitochondria; N, nucleus

Hepatocytes of fish exposed to silver nanoparticles showed glycogen depletion compared to the control (Table 3). Numerous hepatocytes showed malformation and membrane anomalies. In fish exposed to AgNPs and CuNPs, some hepatocytes showed nuclear degradation, heterochromatic nucleoli, karyopyknosis, and necrosis (Figs. 6 and 7).

**Fig. 8:**
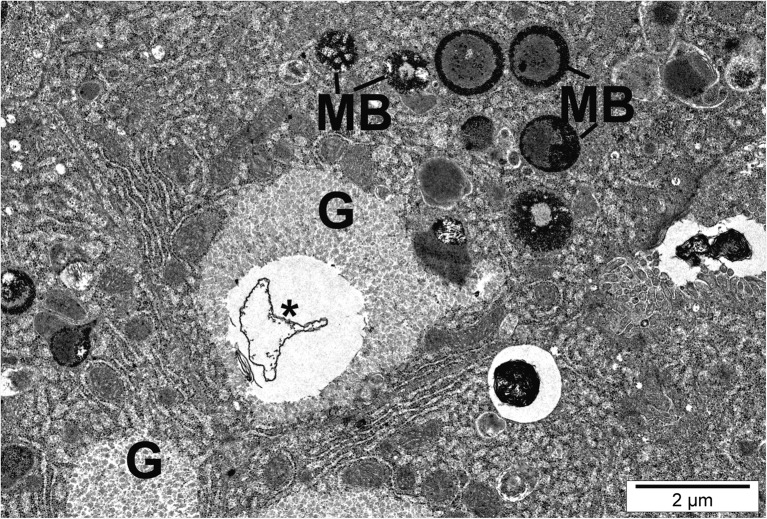
Hepatocyte of fish exposed to copper nanoparticles. Cells with myelin-like bodies (MB) and granular rings showing lipofuscin pigment and vesicular cisternae (*) in glycogen (G) areas

**Fig. 9:**
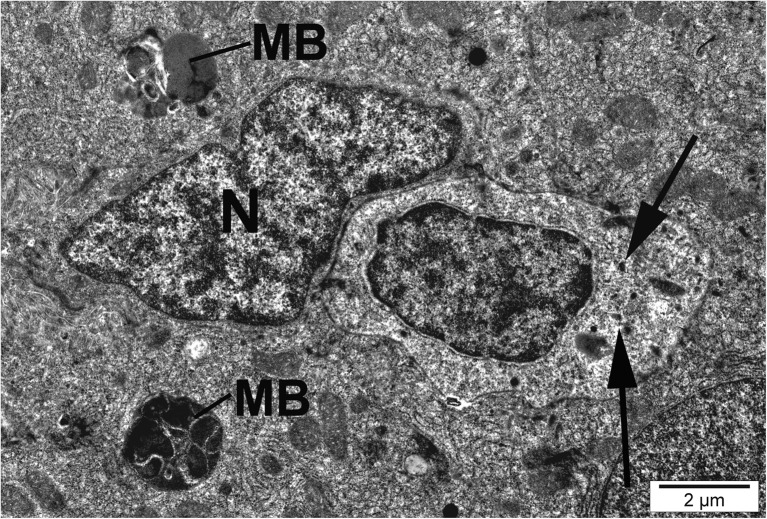
Liver of fish exposed to silver nanoparticles. Myelin-like bodies (MB) and phagosomes with myelin figure deposits and nonmetabolized material indicating cell degeneration and macrophage with nanoparticles (black arrows)

Hepatocytes of fish exposed to AgNPs and CuNPs showed an increase in lysosome abundance, some of them being membranous phagosomes (myelin-like bodies, vacuoles) (Fig. 8). Semithin and ultrathin sections of trout hepatic tissue exposed to AgNPs and CuNPs revealed hypertrophic Kupffer cells of irregular pyknotic nuclei (Table 2 and Fig. 9). Some of them were vacuolated and showed numerous lysosomes, sometimes phagocytic deposits, and small bodies of high electron density, probably nanoparticles (Fig. 9). Nanoparticles visible as black electron-dense spots were observed also in the hepatocyte cytoplasm and mitochondria (Figs. 5 and 7).

## Discussion

Histological and ultrastructural alterations are important biomarkers in toxicological studies. They are often observed in the hepatic tissue of intoxicated fish due to their key role in detoxication. Both in vivo (Govindasamy and Rahuman 2012; Al-Bairuty et al. 2013; Wang et al. 2015; Ostaszewska et al. 2016) and in vitro histopathological studies (Fernández et al. 2013; Connolly et al. 2015; Wang et al. 2016) concerning the effects of various nanoparticles in fish were performed. The liver as the main organ in which biotransformation of toxic agents takes place is a target organ for nanoparticles (Xie et al. 2011). It was proven that AgNPs and CuNPs induced cytotoxic alterations in hepatocytes (Ostaszewska et al. 2016) which resulted in liver damage and accumulation of nanoparticles in the organ (Gaiser et al. 2013) and disrupted hepatic metabolism (Lei et al. 2015).

The results of the present study confirmed earlier reports that the liver was a target organ for AgNPs (Jarrar et al. 2014) and CuNPs (Lei et al. 2015) and the site of their accumulation. Our study revealed that nanoparticles accumulated in the hepatocyte cytoplasm, mitochondria and phagolysosomes, and in Kupffer cells and were visible as black electron-dense spots. Other authors observed nanoparticles in erythrocyte and hepatocyte cytoplasm of common carp exposed to zinc oxide nanoparticles (Lee et al. 2014) and in the hepatocytes of *Danio rerio* (Choi et al. 2010).

Kupffer cells are hepatic tissue macrophages and play an important role in hepatic homeostasis and physiology. They are also involved in the acute and chronic responses of liver to toxic compounds (Winwood and Arthur 1993). Kupffer cells are phagocytes and participate in the first-line defense against nanoparticles (Nishimori et al. 2009). Parenchymal sinusoids of rainbow trout exposed to silver and copper nanoparticles showed hypertrophic Kupffer cells. The high density of Kupffer cells observed in the liver of fish exposed to copper nanoparticles indicates the activation of the immune system. On the other hand, the liver of fish treated with silver nanoparticles probably exhausted its defense potential.

The liver is an important site of glucose metabolism; therefore, carbohydrate storage may be used as indicator of hepatic function. Hepatocytes convert glucose into glycogen, synthesize glucose in the gluconeogenesis process, and release glucose by glycogenolysis (Gharaei et al. 2011). In fish subjected to stress, hepatic and muscle glycogens are used as emergency energy source; therefore, changes in glycogen level may be used as a sensitive indicator of physiological status (Cicik and Engin 2005). In the present study, hepatocytes of fish exposed to AgNPs showed considerably lower glycogen storage compared to the control and fish treated with CuNPs. Similar changes were observed by Almansour et al. (2016) in hepatocytes of rat exposed to nanosilver. The depletion of glycogen indicates that oxidative stress induced by AgNPs may affect the activity of certain enzymes of glycogen synthesis pathway (Almansour et al. 2016).

Statistically higher proliferation index observed in the hepatic parenchyma of fish exposed to copper nanoparticles and the presence of cells condensing glycogen may indicate the phagocytic ability of cells and regenerative potential of damaged hepatocytes.

Our study revealed numerous lysosomes in the hepatocytes of fish exposed to Ag and Cu nanoparticles. According to Lee et al. (2014), this may indicate the activation of biodefense mechanism against nanoparticles. The obtained results indicate that AgNPs and CuNPs cause formation of multilamellar bodies. Lamellar myelin figures were of lysosomal origin and were composed mainly of phospholipids, glucosylceramide, and enzymes of autophagic activity. Formation of myelin-like bodies may indicate pathologic phospholipid properties and cytotoxicity caused by AgNP exposure (Schmitz and Muller 1991; Anderson and Borlak 2006).

In contrast, nonspecific stress response, such as peroxisome proliferation and cytoplasmic myelinated bodies, and formation of intralysosomal myelin-like membrane stacks were observed in the liver of fish exposed to both silver and copper nanoparticles. Peroxisome proliferation and lysosomal inclusions, reduction and degeneration of RER, and excessive lipid storage in hepatocytes were observed by Zahn et al. (1996) during in vitro culture of trout hepatocytes. Morphological alterations similar to those observed in the present study such as disorganization of organelles within cytoplasm, vacuolation, dilatation, and fragmentation of RER were observed in *Brachydanio rerio* exposed to copper sulfate (Paris-Palacios et al. 2000). These authors suggested a nonspecific adaptive response of the liver to stress. According to other authors (Bowen 1984; Wyllie et al. 1984), an increase in number and volume of peroxisomes and lysosomes, mitochondrial matrix swelling, RER dilatation, and disorganization of organelles in the cytoplasm induced by nanoparticles are typical cell reactions indicating necrosis. Dilatation of RER cisternae is considered a result of excessive protein retention due to reduced secretory activity (Paris-Palacios et al. 2000). Degranulation of RER cisternae is another symptom of RER damage, probably due to disturbed protein synthesis (Ghadially 1988). Disorganization of organelles, macrophage infiltration, dilatation and fragmentation of RER, and an increase in the number of lysosomes, autophagosomes, and myelin bodies were observed in the liver of fish exposed to both silver and copper nanoparticles. Cytoplasm degeneration and karyorrhexis indicate that nanoparticles may interact with hepatic enzymes and other proteins which results in oxidative stress and reactive oxygen species (ROS) formation. In turn, ROS may cause hepatocyte apoptosis and necrosis (Choi et al. 2010). The liver plays an important role also in lipid metabolism. Lipid storage results from dietary fat absorption, de novo synthesis of fatty acids in lipogenesis process, and their catabolism during lipolysis. Excessive accumulation of lipid droplets in hepatocytes of fish exposed to AgNPs and CuNPs indicates reduced fatty acid utilization due to their toxicity or impaired lipid catabolic activity of affected cells. Moreover, the exposure to nanoparticles causes lysosomal autophagy which indicates the necessity for degradation of foreign matter and damaged organelles by hepatocytes affected by nanoxenobiotics. Nanoparticle cytotoxicity probably involves oxidation of lipids in subcellular membranes. According to Khatchadourian and Maysinger (2009), assessment of lipid droplet accumulation may be used as a biomarker of oxidative stress and lipid homeostasis. The results of earlier studies showed that nano-TiO_2_ caused hepatic lesions and lipidosis in carp (Hao et al. 2009) and trout (Handy et al. 2008). Accumulation of lipid droplets is probably caused by impairment of mitochondrial β-oxidation of fatty acids due to mitochondrial membrane damage which results in an increase in triglyceride storage (Vickers et al. 2006). Mitochondrial lesions such as edema, cristolysis, curvature, and elongation accompanied by their different sizes and the presence of nanoparticles were observed in the present study. Similar changes were also noticed in rat exposed to AgNPs (Almansour et al. 2016) and CuNPs (Lei et al. 2015). According to Asharani et al. (2009), AgNPs enter the organelles, particularly the mitochondria. Cristolysis induced by AgNPs may affect oxidative phosphorylation and ATP production due to hampering of electron transport (Al Gurabi et al. 2015). Mitochondria play the key role in hepatic metabolism, and obviously, most chronic hepatic diseases are related to mitochondrial disorders. Excessive damage to lysosomal membranes by toxic nanoparticles may result in leakage of hydrolytic enzymes which promotes cell apoptosis and necrosis (Kurz et al. 2008). According to Piao et al. (2011), AgNP cytotoxicity is related to promotion of ROS production and oxidative stress-induced apoptosis. Piao et al. (2011) suggests that AgNPs cause cytotoxicity by oxidative stress-induced apoptosis and damage to cellular components.

The results of the present study showed that silver nanoparticles were more toxic to rainbow trout hepatocytes compared to copper nanoparticles. Despite lower survival and growth rate of fish exposed to copper nanoparticles, histological analysis revealed higher regenerative potential of their hepatocytes, higher glycogen storage ability, higher proliferation index, and higher number of Kupffer cells.
